# Manipulation of Amino Acid Levels with Artificial Diets Induces a Marked Anticancer Activity in Mice with Renal Cell Carcinoma

**DOI:** 10.3390/ijms232416132

**Published:** 2022-12-17

**Authors:** José Manuel Calderón-Montaño, Emilio Guillén-Mancina, Julio José Jiménez-Alonso, Víctor Jiménez-González, Estefanía Burgos-Morón, Alfonso Mate, María Concepción Pérez-Guerrero, Miguel López-Lázaro

**Affiliations:** 1 Department of Pharmacology, Faculty of Pharmacy, University of Seville, 41012 Sevilla, Spain; 2 Department of Physiology, Faculty of Pharmacy, University of Seville, 41012 Sevilla, Spain

**Keywords:** amino acids, cancer, cancer metabolism, cancer therapy, kidney cancer, renal adenocarcinoma, renal cancer, metastasis, selective amino acid restriction therapy, restriction

## Abstract

Targeted therapies with antiangiogenic drugs (e.g., sunitinib) and immune checkpoint inhibitors (e.g., anti-PD-1 antibodies) are the standard of care for patients with metastatic renal cell carcinoma. Although these treatments improve patient survival, they are rarely curative. We previously hypothesized that advanced cancers might be treated without drugs by using artificial diets in which the levels of specific amino acids (AAs) are manipulated. In this work, after showing that AA manipulation induces selective anticancer activity in renal cell carcinoma cells in vitro, we screened 18 artificial diets for anticancer activity in a challenging animal model of renal cell carcinoma. The model was established by injecting murine renal cell carcinoma (Renca) cells into the peritoneum of immunocompetent BALB/cAnNRj mice. Mice survival was markedly improved when their normal diet was replaced with our artificial diets. Mice fed a diet lacking six AAs (diet T2) lived longer than mice treated with sunitinib or anti-PD-1 immunotherapy; several animals lived very long or were cured. Controlling the levels of several AAs (e.g., cysteine, methionine, and leucine) and lipids was important for the anticancer activity of the diets. Additional studies are needed to further evaluate the therapeutic potential and mechanism of action of this simple and inexpensive anticancer strategy.

## 1. Introduction

Kidney cancer is a relatively common cause of disease and death: ~430,000 new cases and ~180,000 deaths were recorded worldwide in 2020 [1]. More than 90% of people diagnosed with kidney cancer have a type of cancer that arises from the renal epithelium and is called renal cell carcinoma [2,3]. Renal cell carcinoma can often be cured if treated when the tumor cells are located in the kidney and immediately surrounding tissue [2]. However, when cancer cells spread beyond the Gerota fascia (a layer of connective tissue that encapsulates the kidneys and adrenal glands) and surgical resection of all metastatic tumors is not possible, renal cell carcinoma generally becomes an incurable disease. Less than 15% of patients with stage IV renal cell carcinoma survive 5 years after diagnosis, and some of these long-term survivors eventually die of the disease [4].

Because renal cell carcinoma is refractory to chemotherapy, the advanced disease has traditionally been treated with interferon-alpha and interleukin-2 [5]. Therapy with these agents achieved responses in only 10–20% of the patients, with durable responses occurring in a subset of responders [6]. Recent understanding of the biology of the disease has led to the discovery of targeted therapies that have overcome the efficacy of these traditional treatments [7,8]. Briefly, tumor growth and survival require angiogenesis (generation of new blood vessels), because the proliferation and survival of the new cancer cells formed during tumor growth depend on the acquisition of nutrients and oxygen through the blood [9]. Activation of the hypoxia-inducible factor (HIF) pathway and subsequent expression of the vascular endothelial growth factor (VEGF) gene are key events in angiogenesis. Renal cell carcinoma is often associated with mutations in the Von Hippel–Lindau (VHL) gene, which result in the up-regulation of HIF, VEGF, and angiogenesis [9]. Therapies targeting the VEGF pathway (and related pathways) have become important components of first-line treatments for patients with metastatic renal cell carcinoma [10,11]. Sunitinib, an orally available angiogenesis inhibitor that targets multiple kinases (VEGFR-1, VEGFR-2, PDGFR, c-Kit) was the first targeted anticancer drug approved for metastatic renal cell carcinoma [6,8]. After sunitinib, other angiogenesis inhibitors have been developed, such as axitinib, pazopanib, and cabozantinib [10,11]. Recent understanding of the mechanisms of immune escape of cancer cells [12] has also allowed the development of new anticancer drugs widely used in renal cell carcinoma. These drugs, called immune checkpoint inhibitors, induce anticancer effects by blocking proteins that inhibit the immune response, including programmed cell death protein 1 (PD-1) and programmed cell death ligand 1 (PD-L1) [12]. Anti-PD-1 (pembrolizumab, nivolumab), anti-PD-L1 (avelumab), and other checkpoint inhibitors are first-line drugs for the treatment of clear cell renal cell carcinoma in combination with angiogenesis inhibitors [13,14,15]. For example, the combination of axitinib plus pembrolizumab versus sunitinib was associated with a longer median progression-free survival (15.1 vs. 11.1 months), a higher objective response (59.3% vs. 35.7%), and a better complete response (5.8% vs. 1.9%), and this combination is a widely used first-line treatment for patients with metastatic renal cell carcinoma [11,13]. However, despite the development of these new treatments, the survival of patients with metastatic disease remains low. It is also important to note that systemic therapies for renal cell carcinoma are very expensive and unaffordable for some patients [16,17]. New therapies are urgently needed for patients with unresectable renal cell carcinoma.

We previously proposed that advanced cancers could be treated without drugs by using artificial diets in which the levels of specific amino acids (AA) are manipulated [18]. Briefly, the aim of this anticancer strategy is to selectively kill cancer cells by exploiting their complete set of DNA alterations through the generation of temporal AA imbalances. Cell survival requires adequate levels of each of the 20 proteinogenic AAs; any cell unable to obtain adequate levels of just one of these AAs will eventually die. The generation of AA imbalances with artificial diets would force cells to activate the genetic programs required to obtain adequate levels of each AA. Normal cells would use their functional genome to adapt to and resist a challenging AA imbalance. Cancer cells would be unable to do so because their DNA alterations would compromise the activation of the genetic programs required to adapt and survive the new environment [18]. The idea is not just to exploit a particular genetic defect involved in the synthesis of a specific amino acid [18,19,20,21,22,23,24,25,26,27,28,29,30]. It is to change the environment under which carcinogenesis occurs, because mutations that provide cancer cells with a survival benefit under a specific environment may cause their death in a different environment [18]. As discussed elsewhere [31,32], evolution and survival depend not only on the acquisition of beneficial DNA changes but also on favorable environments for these DNA changes. Since all cancers have originated and evolved under environments in which the levels and ratios of the 20 proteinogenic AAs are relatively constant, changing these levels and ratios can result in the selective killing of cancer cells. This anticancer strategy has previously been discussed in detail [18].

In this work, we have evaluated the efficacy of this therapeutic strategy in renal carcinoma cells in vitro and in vivo following a patient-oriented approach [33]. After observing selective anticancer activity in vitro, we used mice with peritoneally disseminated renal cell carcinoma to screen 18 artificial diets in which the levels and ratios of selected AAs were manipulated. Several diets showed marked anticancer activity in vivo. Mice treated with one of our diets lived longer than mice treated with sunitinib or anti-PD-1 immunotherapy, two widely used treatments in patients with metastatic renal cell carcinoma. Controlling the levels of several AAs (e.g., cysteine, methionine, and leucine) and lipids was important for the anticancer activity of the diets.

## 2. Results

### 2.1. Selective Amino Acid Restriction Induces Cytotoxicity and Selectivity in Renal Cell Carcinoma Cells In Vitro

**Figure 1:**
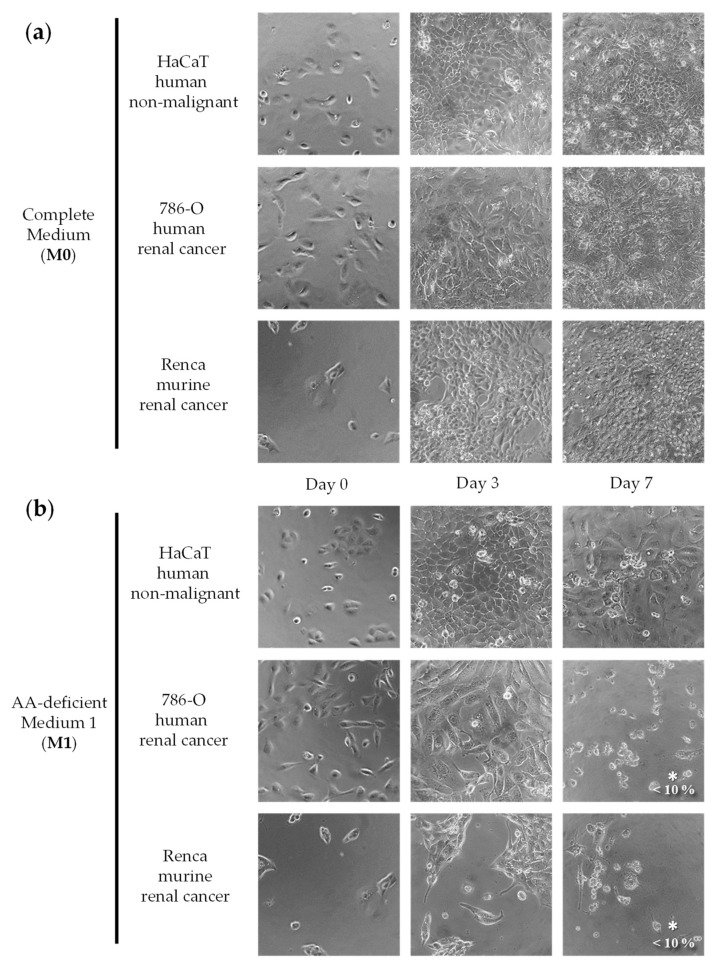
Effect of amino acid restriction on non-malignant cells and renal cell carcinoma cells. Representative photographs at 20× magnification are shown. The percentage of cell viability with respect to cells grown in the complete medium was determined with the MTT assay and is shown at the bottom right of the images when it was less than 10%. For statistical analysis, the Mann–Whitney U test was used to compare the viability of cancer cells (786-O or Renca) versus normal cells (HaCaT); * indicates *p* < 0.05. The composition of Medium 0 (**a**) and Medium 1 (**b**) is shown in Table 1.

**Figure 2:**
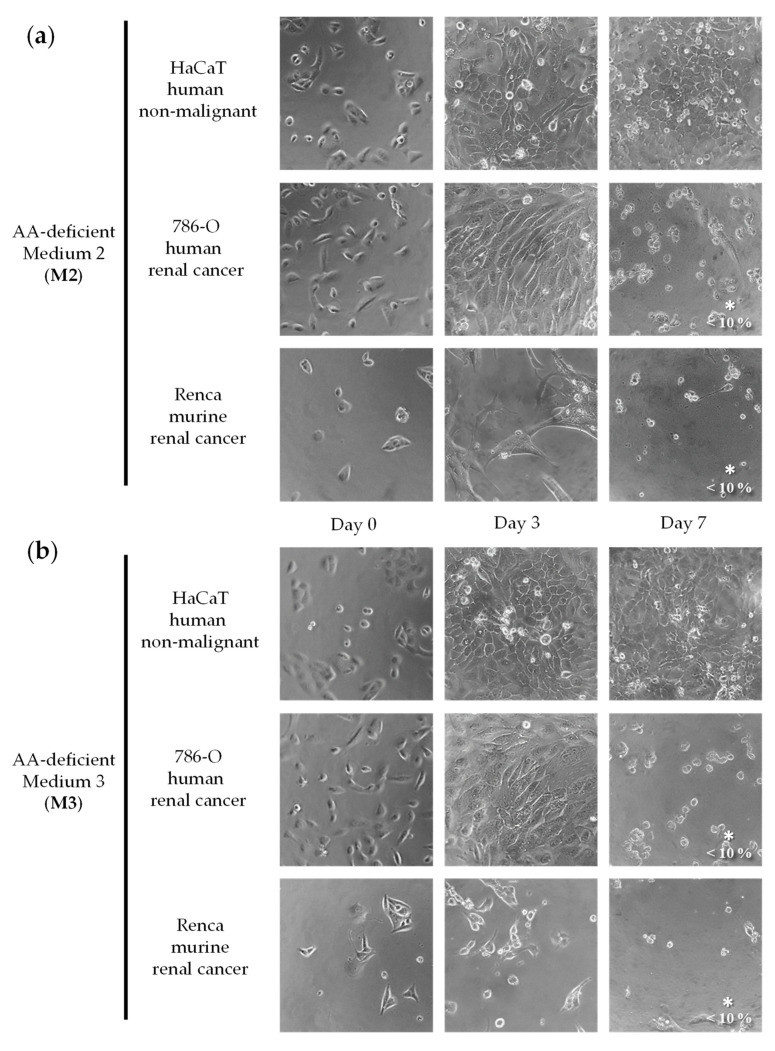
Effect of amino acid restriction on non-malignant cells and renal cell carcinoma cells. Representative photographs at 20× magnification are shown. The percentage of cell viability with respect to cells grown in the complete medium was determined with the MTT assay and is shown at the bottom right of the images when it was less than 10%. For statistical analysis, the Mann–Whitney U test was used to compare the viability of cancer cells (786-O or Renca) versus normal cells (HaCaT); * indicates *p* < 0.05. The composition of Medium 2 (**a**) and Medium 3 (**b**) is shown in Table 1.

**Figure 3:**
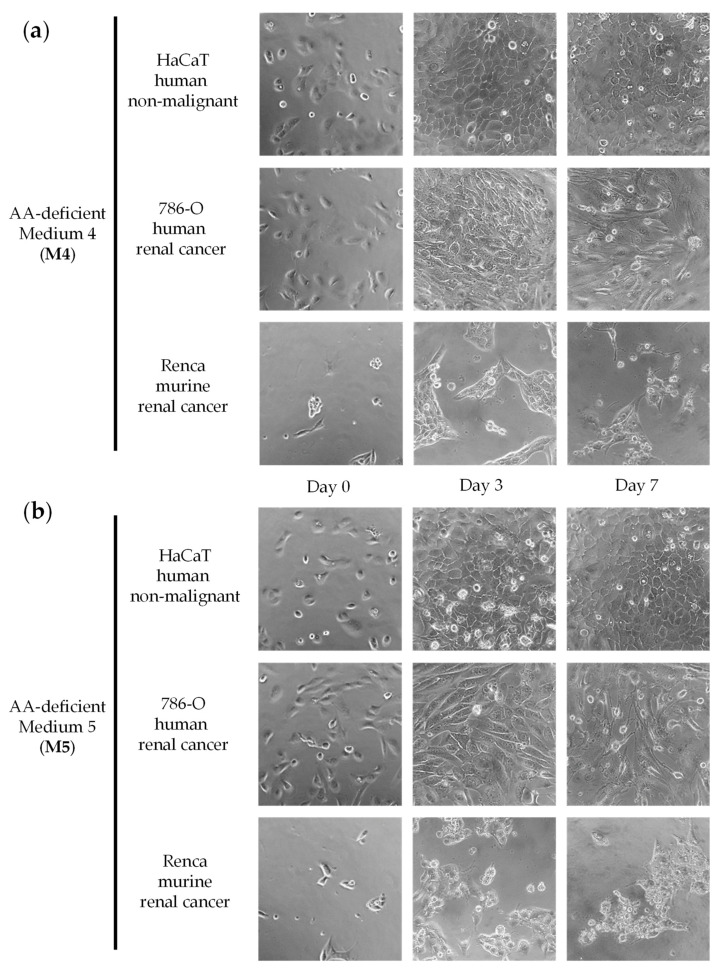
Effect of amino acid restriction on non-malignant cells and renal cell carcinoma cells. Representative photographs at 20x magnification are shown. The percentage of cell viability with respect to cells grown in the complete medium was determined with the MTT assay and is shown at the bottom right of the images when it was less than 10%. For statistical analysis, the Mann–Whitney U test was used to compare the viability of cancer cells (786-O or Renca) versus normal cells (HaCaT). The composition of Medium 4 (**a**) and Medium 5 (**b**) is shown in Table 1.

**Figure 4:**
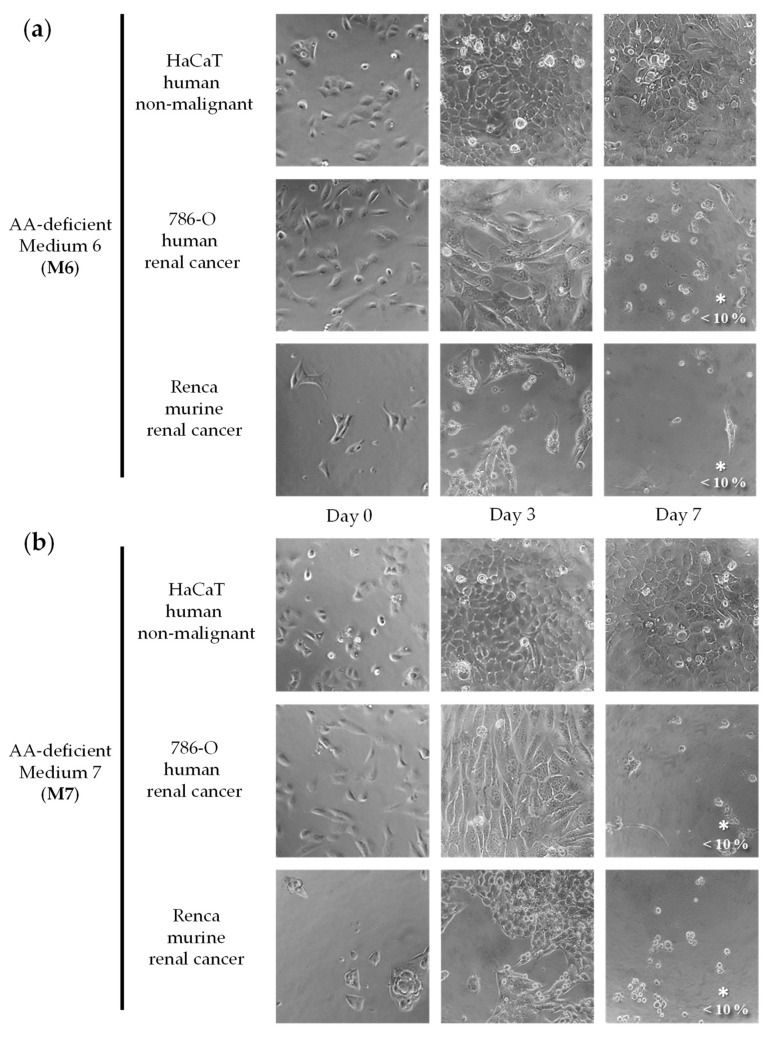
Effect of amino acid restriction on non-malignant cells and renal cell carcinoma cells. Representative photographs at 20× magnification are shown. The percentage of cell viability with respect to cells grown in the complete medium was determined with the MTT assay and is shown at the bottom right of the images when it was less than 10%. For statistical analysis, the Mann–Whitney U test was used to compare the viability of cancer cells (786-O or Renca) versus normal cells (HaCaT); * indicates *p* < 0.05. The composition of Medium 6 (**a**) and Medium 7 (**b**) is shown in Table 1.

We initially screened a variety of AA-manipulated media in murine renal cancer cells (Renca) and in non-malignant human epithelial cells (HaCaT) to detect cytotoxicity and selectivity. We focused on restrictions of non-essential amino acids (NEAA) because essential amino acids (EAA) cannot be synthesized by humans and their restriction can lead to non-selective cytotoxicity [18]. We tested our AA-manipulated media at several exposure times and recovery periods, and we used a microscope to visualize cell numbers and shapes daily. Cytotoxicity and selectivity were observed in most cases at specific exposure times. Although some combinations of AAs were more cytotoxic and selective than others, we did not observe a clear relationship between the elimination of specific AAs and the selectivity against the cancer cell line. Based on this initial screening, we selected seven AA-manipulated media for further study. The cytotoxicity and selectivity of the seven AA-manipulated media (Table 1; Materials and Methods) were evaluated in murine renal cancer cells (Renca), human renal cancer cells (786-O), and non-malignant human epithelial cells (HaCaT) (Figure 1, Figure 2, Figure 3 and Figure 4). Cells were grown in these AA-deficient media for seven days. Cells were monitored by microscopic visualization and photographed on days 3 and 7. On day 7, cell viability was also estimated with the MTT assay.

**Figure 5:**
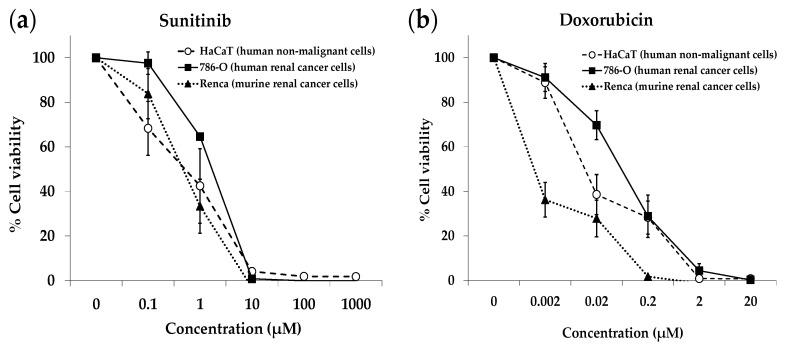
Cytotoxicity of sunitinib (**a**) and doxorubicin (**b**) against human renal cancer cells, human non-malignant cells, and murine renal cancer cells. The cells were exposed to the drugs for 72 h. Cell viability was then assessed with the MTT assay. Data show the mean ± SEM of at least 3 independent experiments.

The results, shown in Figure 1, Figure 2, Figure 3 and Figure 4, indicate that several AA-manipulated media were more cytotoxic against murine and human renal cancer cells than against human non-malignant cells. Non-malignant HaCaT cells incubated with our AA-deficient media proliferated slower than those incubated with complete media, but they divided and eventually saturated the wells. HaCaT cells were also able to transform the MTT compound into the colored formazan product in all cases, therefore indicating that these non-malignant cells were viable. Clear cytotoxic effects were observed for human and murine cancer cell lines placed for 7 days in 5 of the 7 artificial media (Figure 1, Figure 2, Figure 3 and Figure 4). On day 3, human renal carcinoma cells (786-O) were less affected than murine renal carcinoma cells (Renca). On day 7, however, cell viability (estimated with the MTT assay) was marginal in both cancer cell lines in 5 of the 7 AA-manipulated media. These data indicate that selective restriction of amino acids induces cytotoxicity and selectivity in renal carcinoma cells in vitro. Less selectivity was observed when the three cell lines were treated for 72 h with the anticancer drugs sunitinib or doxorubicin (Figure 5). The IC50 value (mean ± SEM, µM) for sunitinib was 1.850 ± 1.633 in HaCaT cells, 1.280 ± 0.023 in 786-O cells, and 0.600 ± 0.251 in Renca cells. The IC50 value (mean ± SEM, µM) for doxorubicin was 0.061 ± 0.051 in HaCaT cells, 0.101 ± 0.049 in 786-O cells, and lower than 0.002 in Renca cells.

### 2.2. Selective Amino Acid Restriction Therapy Improves Survival in Mice with Renal Cell Carcinoma

Patients with localized renal cell carcinoma can often be cured by surgery, while those with disseminated disease lack curative therapies. Therefore, we used mice with disseminated renal cancer cells to screen the anticancer activity of our treatments. Immunocompetent BALB/cAnNRj mice were intraperitoneally injected with 100,000 Renca cells. If left untreated, 100% of the mice develop numerous tumors and die approximately 1 month later. Treatments started eight days after injecting these metastatic cancer cells. Because this animal model is highly reproducible, and to follow Animal Ethics Committee recommendations, we initially used 2–4 mice per group in most cases to limit the number of animals to a minimum. After this initial screening, we repeated the experiments using more mice to confirm the activity of the most active diet.

**Figure 6:**
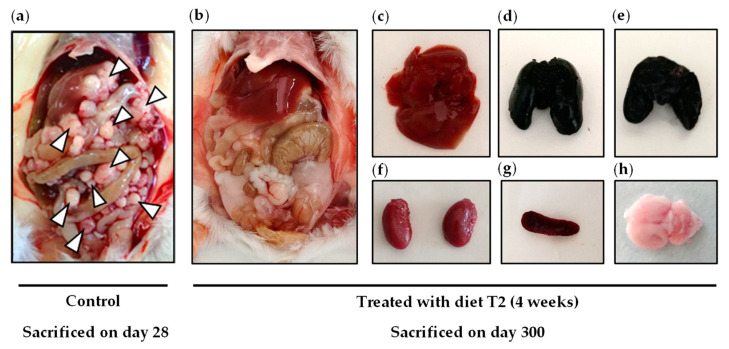
Autopsy images of an untreated mouse (control) and a mouse treated with diet T2. Tumors in the peritoneal cavity of control mice sacrificed on day 28 (**a**). Lack of tumors in the peritoneal cavity (**b**), liver (**c**), lungs (front view, dyed with India ink) (**d**), lungs (back view, dyed with India ink) (**e**), kidneys (**f**), spleen (**g**), and brain (**h**) in the mouse treated with T2 sacrificed on day 300.

We initially evaluated the in vivo anticancer activity of diet T1 (equivalent to culture medium M1) and diet T2 (equivalent to culture medium M2). Diet T1 lacks ten NEAAs; it contains the nine EAAs and Gln. Diet T2 lacks six NEAAs; it contains the nine EAAs, Gln, Arg, Gly, Asp, and Ala. The detailed compositions of diets T1 and T2 are shown in Table 2 (Materials and Methods). Animals fed a normal diet (control group, *n* = 4) developed symptoms of advanced disease and were sacrificed on days 25, 28, 28, and 29 after the injection of the cancer cells. Autopsies confirmed the presence of numerous tumors in the peritoneal cavity that affected several organs. Survival of mice treated with diet T1 (*n* = 2) was somewhat higher; these mice lived 28 and 38 days. Mice fed diet T2 (*n* = 2) lived longer than untreated mice; one mouse lived 42 days and the other mouse was sacrificed on day 300 without finding any tumor on autopsy (Figure 6).

**Figure 7:**
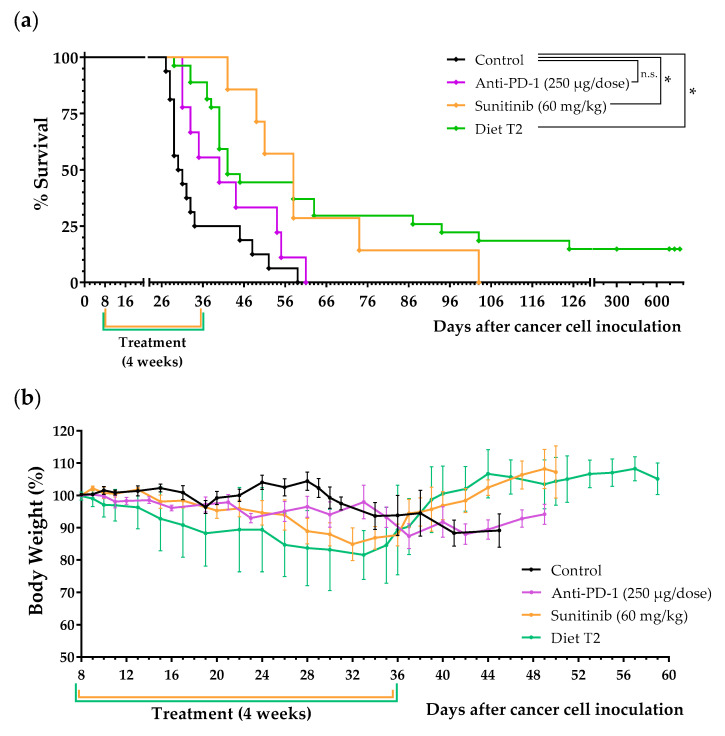
Anticancer effect of diet T2, sunitinib, and anti-PD-1 in mice with renal cell carcinoma. Survival (**a**) and body weight (**b**) of mice treated with sunitinib (60 mg/kg/day, 28 days), with anti-PD-1 (250 µg/dose, every 4 days for a total of 4 doses), with diet T2 (28 days), or left untreated (control). Treatments began 8 days after the intraperitoneal injection of 100,000 Renca cancer cells. Statistically significant differences were found for sunitinib and diet T2 versus control. Data were pooled from several independent experiments. Statistical analysis was calculated using the Gehan–Breslow–Wilcoxon test; n.s. indicates non-significance (*p* > 0.05), * indicates significance (*p* < 0.05). Body weights are expressed as percentages relative to the body weight at the beginning of the treatments (day 8).

After this initial screening, we performed several independent experiments to confirm the in vivo anticancer activity of diet T2 and to compare it with the activity of sunitinib and an anti-PD-1 antibody (similar to nivolumab), two treatments widely used in patients with metastatic renal cell carcinoma. These treatments were administered under clinically relevant conditions (at high doses under an administration protocol similar to that used in cancer patients). Mice receiving the same treatment are merged in the survival curves to facilitate comparison between groups (Figure 7 and Table 3). The results revealed that most of the animals fed diet T2 lived longer than the untreated mice. Sunitinib treatment clearly stopped disease progression during the first 3 weeks of treatment. However, small abdominal tumors were palpable during the fourth week. The disease started to progress very quickly 3–4 days after sunitinib withdrawal, and in many cases, there was no time to resume treatment according to the standard 4 weeks on, 2 weeks off (4/2) schedule. Sunitinib clearly improved mice survival, but autopsies revealed that tumor loads in these animals were eventually similar to those in untreated mice. Anti-PD-1 treatment also delayed the progression of the disease; however, it showed a modest increase in survival. Most mice fed our AA-manipulated diet lived longer than mice treated with the anti-PD-1 antibody, and approximately 25% of mice fed T2 lived longer than mice treated with sunitinib. Importantly, four mice treated with diet T2 (from three independent experiments) lived very long (Figure 7a). As mentioned in the previous paragraph, one of the mice was sacrificed on day 300 and no tumors were found on autopsy (Figure 6). Another mouse died on day 695 (probably from natural causes) and no tumors were found on autopsy. The other two mice were sacrificed on days 734 and 774 and one tumor was found in the lungs of each mouse; however, we cannot assure that these tumors were caused by the cancer cells inoculated 2 years earlier. Mice fed diet T2 showed a progressive, but reversible, loss of body weight during treatment. A similar effect was also observed in mice treated with sunitinib (Figure 7b). The weights of the spleen and liver of animals treated with diet T2 or with anti-PD1 immunotherapy were higher than those of untreated animals. Sunitinib-treated animals showed the lowest spleen and liver weights (Figure S1). No long-term toxicity was observed in mice on diet T2 that survived treatment. Supplementary Video S1 shows the appearance and behavior of an untreated mouse and a mouse treated with diet T2 (labeled SAART) on day 28.

### 2.3. Screening for In Vivo Anticancer Activity of 10 Diets Based on Diet T2

**Figure 8:**
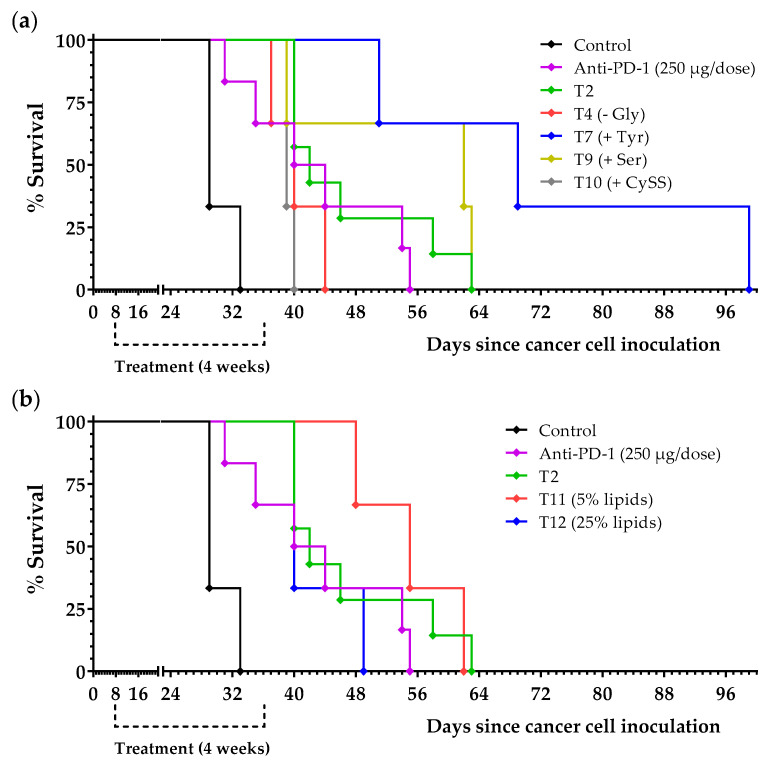
Survival of mice with kidney cancer treated with diets based on diet T2. Treatments started 8 days after cancer cell inoculation. Mice were treated with anti-PD-1 (250 µg/dose, every 4 days for a total of 4 doses), with an artificial diet for 28 days (the normal diet was replaced by one of the artificial diets specified in the figures), or were left untreated (control, normal diet). In these artificial diets (see composition in Table 2), NEAAs were added or eliminated with respect to diet T2 (**a**), or lipid levels were reduced or increased with respect to diet T2 (**b**). See the main text for further details.

Because diet T2 showed marked anticancer activity in vivo, we prepared 10 new diets based on diet T2 to screen their anticancer activity in mice with renal cancer. In these diets (see Table 2), NEAAs were added or eliminated with respect to diet T2. Most mice fed these T2-derived diets lived longer than untreated mice (Table 4 and Figure 8a). The elimination of Ala and Asp (T3) or Gly (T4), and the addition of Glu (T8) or CySS (T10) resulted in diets that were less effective than diet T2. The addition of Pro (T6) or Asn (T5) resulted in diets with similar efficacy to diet T2. The addition of Tyr (T7) or Ser (T9) increased mice survival compared to diet T2. Note that, in this experiment, all mice on diet T2 eventually died. Although diet T7 showed high anticancer activity in this experiment, two additional experiments revealed that the addition of Tyr to the T2 diet resulted in moderate anticancer activity (all animals eventually died). Survival (means ± SEM values) was 28.9 ± 1.0 days for untreated animals and 45.9 ± 7.1 days for mice on diet T7 (Figure S2).

Eight days after cancer cell inoculation, the animals were treated with anti-PD-1 antibody (250 μg administered intraperitoneally on days 8, 12, 16, and 20), with diet T2, with diets based on diet T2 (T3–T12), or were left untreated (control, normal diet). The *p*-value was calculated with the Gehan–Breslow–Wilcoxon test. Diet compositions are shown in Table 2. See the main text for further details.

Since lipid metabolism plays a key role in renal cell carcinoma [34,35], we also evaluated the anticancer activity of two T2-derived diets in which lipid levels were reduced to 5% (diet T11) or increased to 25% (diet T12); diet T2 has 14% lipids (Table 2). Diet T11 was better than diet T2, and diet T12 was worse than diet T2, indicating that the reduction in lipid levels can increase the anticancer activity of the artificial diets (Table 4 and Figure 8b).

### 2.4. Methionine, Cysteine, and Leucine Levels Are Important for In Vivo Anticancer Activity

Although diet T2 showed striking anticancer activity in mice with renal cell carcinoma (Figure 7a, Table 3), mice fed this diet lost weight during treatment (Figure 7b). To overcome this possible limitation, we prepared new artificial diets that incorporate small amounts of the protein casein. This protein provides low levels of the sulfur-containing AAs Cys and Met [36]. Previous studies have shown conflicting results regarding the levels of Met required to achieve in vivo anticancer activity. Although some studies indicate that Met restriction induces anticancer activity [30,37], others not only show that Met restriction promotes tumor growth, but also that Met supplementation induces anticancer activity [38,39]. We, therefore, prepared several casein-based diets with low and high levels of Met (diets T13–T16). Since Cys levels can condition Met requirements [40,41], we also controlled the levels of Cys in these diets. In addition, we prepared a casein-based diet supplemented with Leu (diet T17), because this AA may prevent proteolysis and weight loss under conditions of AA restriction [42,43]. In all these diets, we further reduced the lipid content to 1%, and we used salmon oil as the source of lipids to ensure sufficient levels of essential fatty acids in our diets.

**Figure 9:**
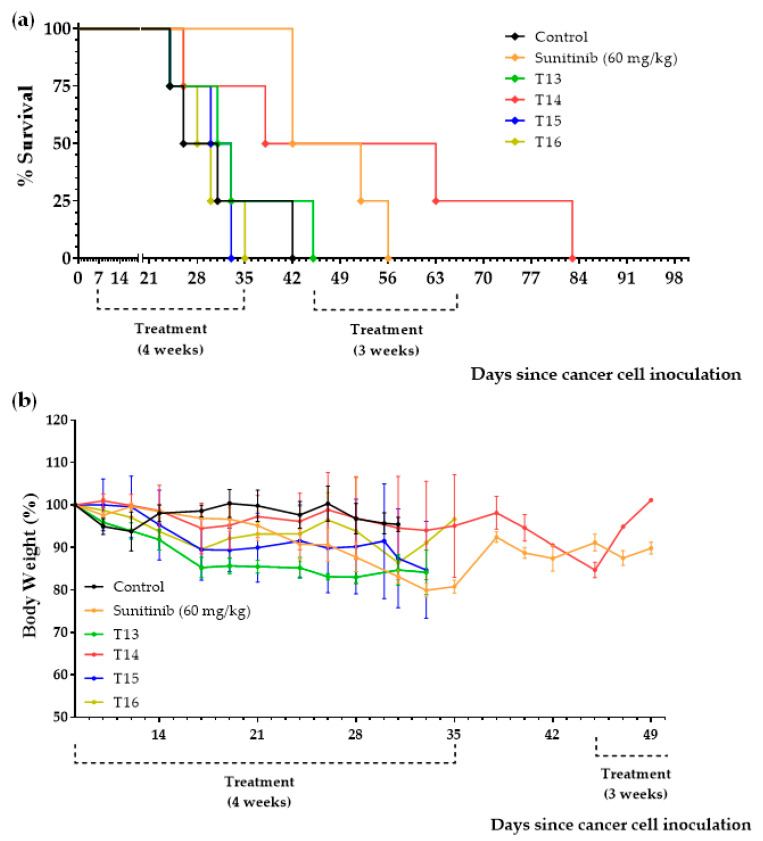
Effect of Cys and/or Met supplementation on the survival (**a**) and body weight (**b**) of mice with renal cell carcinoma treated with casein-based diets. Diet T13 contains 6% casein, Diet T14 contains 6% casein + 0.5% Cys, Diet T15 contains 6% casein + 0.5% Met, and Diet T16 contains 6% casein + 0.5% Cys + 0.5% Met. See the main text for details.

**Figure 10:**
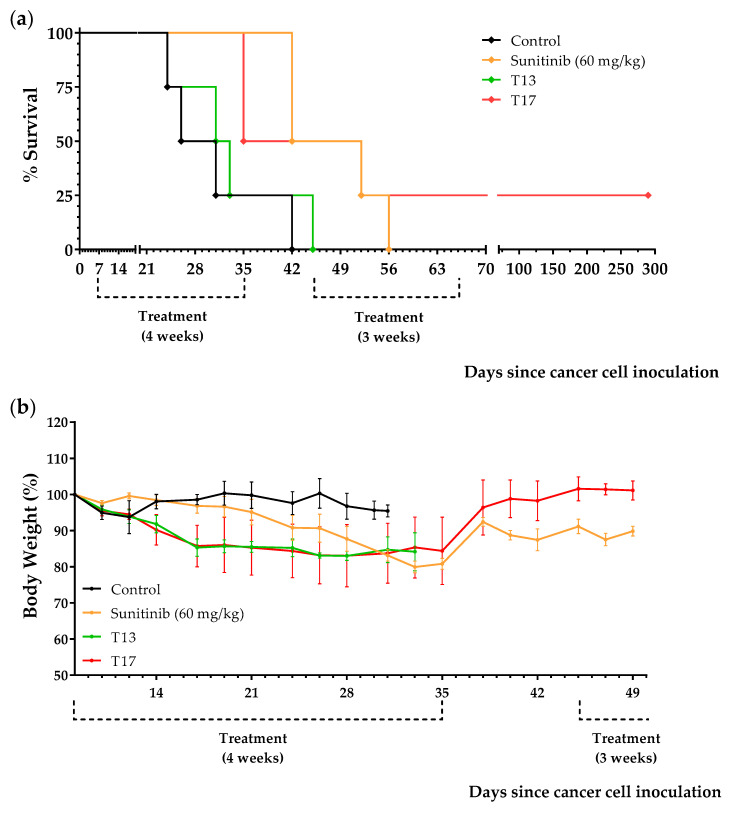
Effect of Leu supplementation on the survival (**a**) and body weight (**b**) of mice with renal cell carcinoma treated with casein-based diets. Diet T13 contains 6% casein, Diet T17 contains 6% casein + 2.5% Leu. See the main text for details.

Treatments began 7 days after the inoculation of 150,000 Renca cancer cells into the peritoneum of immunocompetent BALB/c mice. Animals were treated with sunitinib (60 mg/kg/day), with diets T13, T14, T15, T16, or T17 (the normal diet was replaced by one of these diets), or were left untreated (control group). Mice that survived the initial 4-week treatment with sunitinib or with the diets were put on a normal diet for 10 days. Then, treatments were restarted for an additional 3-week period. The results, shown in Table 5 and Figure 9 and Figure 10, indicate that diets T14 and T17 induced a marked anticancer activity in mice with renal cell carcinoma; mean survivals were higher than for sunitinib. Supplementing the diets with 0.5% Met abolished the activity of the diets (diets T15 and T16 were completely ineffective; note that these diets contain approximately 0.67% Met: 0.5% from the Met supplement + 0.17% from the 6% casein). However, Met restriction was not sufficient to obtain high anticancer activity (diet T13 had mild anticancer activity). When diet T13 (whose only source of AAs is 6% casein) was supplemented with either 0.5% Cys (diet T14) or 2.5 Leu (diet T17), marked in vivo anticancer effects were observed. One mouse fed T17 survived treatment and was sacrificed on day 290 without any symptoms of disease. However, the autopsy revealed the presence of a metastic tumor in the lungs (Figure S3).

**Figure 11:**
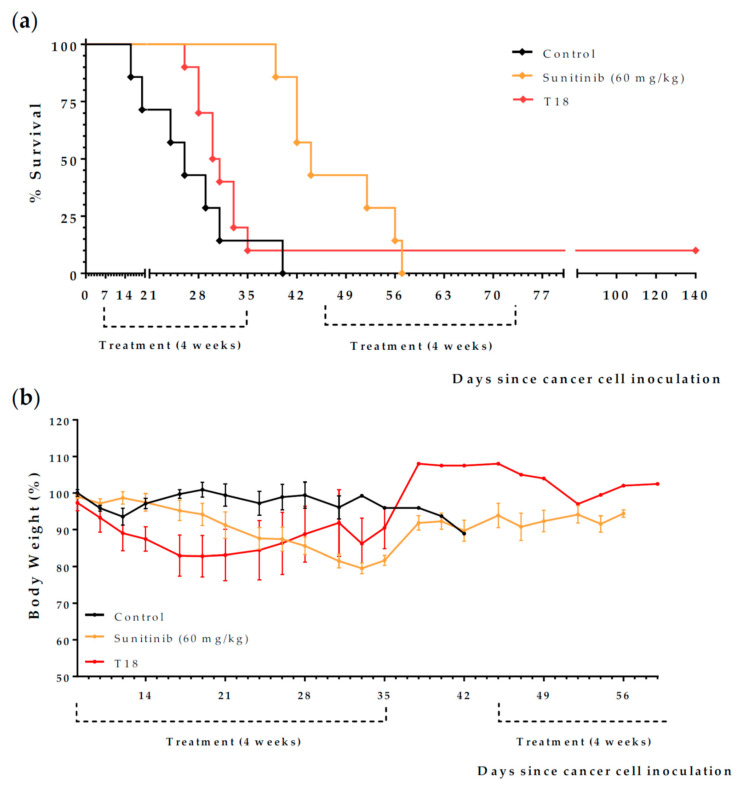
Anticancer activity of diet T18 and sunitinib in mice with renal cell carcinoma. (**a**) Survival of the mice left untreated (control), treated with diet T18 (normal diet was replaced with this diet), or treated with oral sunitinib (60 mg/kg/day). (**b**) Body weights (mean percentage ± SEM) relative to the body weights at the start of the treatments (day 7). Data were pooled from two independent experiments.

Finally, we evaluated the anticancer activity of diet T18, which consisted of adding 5% Gln to diet T17 (Table 2); Gln is involved in the cellular transport of Leu and in the biosynthesis of NEAA [44]. We have recently reported that diet T18 (also known as TC5) induces a marked anticancer activity in mice with metastatic colon cancer [45]. Treatments began 7 days after the inoculation of 150 000 Renca cancer cells into the peritoneum of immunocompetent BALB/c mice. Animals were left untreated (control group, *n* = 7), or were treated with sunitinib (60 mg/kg/day, *n* = 7) or diet T18 (*n* = 10). Mice that survived an initial 4-week treatment were put on a normal diet for 10 days. Then, treatments were restarted for an additional 4-week period. Although the standard treatment sunitinib was better than diet T18 (Figure 11 and Table 6), one mouse fed T18 survived treatment and was sacrificed on day 140 without finding any sign of tumors in the autopsy.

## 3. Discussion

Patients with metastatic renal cell carcinoma need new treatments; current therapies prolong patient survival but are rarely curative. Targeting the metabolic alterations of cancer cells is a promising strategy for the development of new treatments [46]. Cancer cells are known to develop a wide range of metabolic adaptations to support their survival and growth, including alterations in the metabolism of AAs [47,48]. Renal cell carcinoma shows alterations in a variety of AAs [49,50], including Arg [51,52], Cys [34,53], and Gln [54,55]. Dietary restriction of AAs has already shown activity in a wide variety of cancer types [19,20,21,22,23,24,25,26,27,28,29,30,56,57]. The aim of this work was to evaluate whether the manipulation of AA levels with artificial diets has therapeutic potential for renal cell carcinoma.

We began our investigation by testing the selective cytotoxicity against cancer cells of several artificial media lacking different AAs. Our in vitro screening revealed that several of our AA-manipulated media induced selective cytotoxicity toward renal cancer cells (Figure 1, Figure 2, Figure 3 and Figure 4). Non-malignant HaCaT cells incubated with our AA-deficient media survived treatment. In contrast, the viability of human and murine renal cancer cells (786-O and Renca cells) was markedly reduced when grown in most of our AA-deficient media, especially M1 and M2 (Figure 1 and Figure 2). The anticancer agents sunitinib and doxorubicin did not show selective cytotoxicity toward human renal cancer cells; their cytotoxicity against normal HaCaT cells was similar to their cytotoxicity against 786-O cancer cells (Figure 5). Mechanistically, media without Cys (M1, M2, M3, M6, and M7) were more cytotoxic and selective than media with Cys (M4 and M5), which suggests that Cys restriction was important for the in vitro anticancer activity of our artificial media (Figure 1, Figure 2, Figure 3 and Figure 4). Cancer cells are known to produce higher levels of reactive oxygen species (ROS) than normal cells [58], and Cys is necessary to generate glutathione (GSH), which in turn is crucial for protecting cells from the cytotoxic activity of ROS. Our in vitro results should be interpreted cautiously, however, because the metabolic environment of cancer cells cultured in vitro is very different from the metabolic environment of cancer cells in vivo [59].

Because in vitro experimental models cannot recapitulate the complex metabolic environment of cancer cells in vivo, we used an in vivo model of renal cell carcinoma to further evaluate our anticancer strategy. This is a challenging in vivo model in which mice are rarely cured when treated with standard drugs used in patients with renal cell carcinoma. We initially prepared diets T1 and T2, which lack the same AAs as in media M1 and M2. Our initial in vivo screening showed that diet T1 (a diet lacking 10 AAs) had modest anticancer activity; one of the two mice treated with this diet lived approximately 10 days longer than untreated mice. Both mice treated with diet T2 (a diet lacking six AAs) lived longer than control mice; one mouse lived two weeks longer than untreated mice, and the other mouse was apparently cured (it was sacrificed on day 300 and no tumors were found on autopsy; Figure 6). We then carried out several independent experiments to confirm this finding. Mice also received two treatments widely used in patients with metastatic renal cell carcinoma: sunitinib and anti-PD-1 immunotherapy. These treatments were administered under clinically relevant conditions (at high doses under an administration protocol similar to that used in cancer patients). The results revealed that the mean survival of mice treated with diet T2 was higher than the survival of mice treated with sunitinib or with anti-PD-1 immunotherapy (Table 3, Figure 7). Like most patients receiving sunitinib or anti-PD-1 immunotherapy, mice receiving these treatments lived longer but eventually died. Importantly, four mice treated with diet T2 had very long survivals; they died or were sacrificed on days 300, 695, 734, and 774. This artificial diet was well tolerated, although most mice suffered reversible weight loss during treatment. No long-term toxicity was observed in the four mice that survived treatment with diet T2.

Because diet T2 showed marked anticancer activity in vivo, we prepared 10 new diets based on diet T2 to try to understand its mechanism of action and improve its anticancer activity. Diet T2 lacks six NEAAs (i.e., Cys, Ser, Tyr, Pro, Asn, and Glu); therefore, six of the ten new diets consisted of the T2 diet supplemented with one of these six AAs (Table 2). In two additional diets, instead of supplementing with an AA, we eliminated Gly or Ala + Asp from diet T2. In the remaining two diets, the lipid content of diet T2 was either reduced to 5% or increased to 25%. These 10 diets were screened in our model of renal cell carcinoma. A group of mice was also treated with anti-PD-1 immunotherapy. As shown in Table 4, all diets improved mice survival, but important differences in activity were observed. Diet T7, which was diet T2 supplemented with Tyr, showed the highest activity in this screening; however, additional experiments revealed that the addition of Tyr did not result in a diet better than diet T2 (diet T2 supplemented with Tyr did not cure any mouse; Figure S2). Previous research showed that double dietary restriction of Tyr and Phe induced anticancer activity [60]. In contrast, our data show that the presence of Tyr and Phe in our diets is not an obstacle to obtaining anticancer activity in mice with renal cell carcinoma. Previous studies have shown that Ser deprivation induces anticancer activity [22]. However, our results indicate that Ser deprivation is not necessary for anticancer activity in renal cancer; mice fed diet T9, which contains Ser, lived longer than mice treated with diet T2, which does not contain Ser (Table 4, Figure 8). Another study indicated that the elimination of both Ser and Gly was important for antitumor activity [21]. However, our data in mice with renal cell carcinoma show that diet T4 (which lacks both Ser and Gly), is worse than diets T9 (which contains both Ser and Gly) and T2 (which contains Gly but lacks Ser). Elimination of Ala and Asp in T2 (diet T3) had a negative effect on the anticancer activity. Both AAs participate in many transamination reactions and their presence may allow healthy cells to synthesize the NEAAs eliminated in diet T2. Supplementation with cystine (a dimer of Cys) (T10) or Glu (T8) also reduced the anticancer activity of the diets. Both Cys and Glu are components of the tripeptide GSH (Glu-Cys-Gly). As mentioned previously, cancer cells have increased ROS levels, and GSH is crucial for protecting cells from the cytotoxic activity of ROS. Cys deprivation is already known to induce anticancer activity in renal cell carcinoma [53]. Finally, diet T11 (diet T2 with 5% lipids) was better than diet T2 (14% lipids), while diet T12 (diet T2 with 25% lipids) was worse than diet T2 (see Table 4 and Figure 8). Lipid metabolic reprogramming and lipid accumulation are hallmarks of clear cell renal cell carcinoma [34,35,61]. Reducing the lipid content of our diets may, therefore, be important to increase their anticancer activity.

Although diet T2 showed marked anticancer activity in vivo (Figure 7, Table 3), mice suffered progressive weight loss during treatment (Figure 7b). Although reversible, this weight loss could be challenging for a fraction of cancer patients who may already have poor nutritional status due to cachexia and the side effects of chemotherapy [62]. In addition, the poor organoleptic properties of diets based on free AAs [37,63,64] may also limit their clinical use. To overcome these possible problems, we continued our investigation by taking a different approach. Instead of using mixtures of free AAs to completely eliminate NEAAs, we used low concentrations of a protein to reduce the levels of all AAs. If necessary, selected free AAs can be supplemented to increase the amount provided by the protein. Since our in vitro and in vivo experiments indicate that Cys restriction is important for activity, we selected the protein casein, which contains very low levels of Cys [36]. After observing that 6% was the smallest percentage of casein required to avoid weight loss in mice (results not shown), we prepared five casein-based diets (diets T13-T17). In all these diets, we reduced lipid levels to 1%, and we selected salmon oil to ensure sufficient levels of essential fatty acids. The levels of Cys, Met, and Leu were the only differences among these five diets (see Table 2). All of these diets, and the first-line drug sunitinib, were tested in our model of renal cell carcinoma. The results, shown in Table 5, revealed that diets T14 and T17 markedly improved mice survival; mean survivals achieved with these diets were higher than the observed in mice treated with sunitinib. The two diets containing the Met supplement (diets T15 and T16) were completely inactive, but Met restriction was insufficient for activity (diet T13 showed low activity). The addition of 0.5% Cys (diet T14) or the addition of 2.5% Leu (diet T17) to the casein-only diet (diet T13) markedly increased the activity. Diet T17 induced the highest anticancer activity, but mice on this diet suffered weight loss during treatment (Figure 10). Diet T14 also induced a marked anticancer activity without causing weight loss in the animals (Figure 9). Diet T18, which induced a high anticancer activity in mice with metastatic colon cancer (diet TC5) [45], also showed activity in mice with renal cell carcinoma (Table 6 and Figure 11). However, only 1 of the 10 mice treated with diet T18 showed a remarkable anticancer response.

The precise mechanism involved in the in vivo anticancer activity of our artificial diets is unknown. However, the screening of 18 diets in mice with renal cancer under similar experimental conditions allows us to draw several general conclusions, which may also be relevant to other types of cancer. The first and most important conclusion is that restriction of a particular AA can have a positive or negative effect on anticancer activity depending on the levels of other AAs. Cys is the clearest example to illustrate this conclusion. In vitro experiments with media M1–M7, and in vivo experiments with diets formulated with free AAs (diets T1–T12), showed that Cys restriction was crucial for activity. However, when we restricted Cys by using casein-based diets, we observed that the effect of Cys on the anticancer activity of the diets was much more complex than previously thought. Mice treated with diet T13, which contains a very small amount of Cys (provided by 6% casein; approximately 0.042% Cys), did not live much longer than untreated mice. Unexpectedly, when we supplemented diet T13 with 0.5% Cys to obtain diet T14, the activity of the diet was markedly improved (diet T14 was better than sunitinib), which indicated that Cys supplementation was important for the activity. However, the positive effect of supplementing 0.5% Cys was completely abolished when the diet was also supplemented with 0.5% Met (diet T16). In addition, the anticancer activity of diets T17 and T18 (which lack the Cys supplement), indicates that supplementing 0.5% Cys was not necessary for activity if the diet is supplemented with 2.5% Leu.

Our second general conclusion is that the anticancer activity achieved by changing the levels of an AA or a group of AAs depends on the levels of other dietary components, such as lipids. For example, the activity of diet T2 can be increased or decreased by changing the percentage of lipids (Figure 8). Since lipids are necessary for cancer development and survival [35,65], reducing lipid levels may have an additive and independent anticancer effect on the anticancer activity achieved by manipulating AAs. However, the anticancer activity of lipid restriction may also depend on the restriction of specific AAs. For example, dietary Met restriction decreases liver stearoyl-CoA desaturase, an enzyme that converts saturated fatty acids into monounsaturated fatty acids [41,66,67]. Dual dietary restriction of Met and monounsaturated fatty acids may cause toxicity in cancer cells by altering the balance between saturated and unsaturated fatty acids [66]. This may contribute to explaining why Met supplementation completely blocked the activity of casein-based diets, which contain a very low amount of fatty acids (their lipid content is 1%). Naturally, this mechanism would not explain the high anticancer activity of diet T2, which contains 0.6% Met and 14% extra virgin olive oil (which is rich in the monounsaturated fatty acid oleic acid). This leads to the last conclusion: our active artificial diets may not share the same mechanism of action. Although the precise mechanism of action is unknown, our artificial diets probably create unfavorable metabolic environments for the proliferation and survival of renal cancer cells. The DNA aberrations that provide cancer cells with a survival advantage under a normal metabolic environment may become a liability under the new metabolic environments created with our artificial diets.

Understanding the precise mechanism by which our artificial diets induce anticancer activity in vivo will be a very complex task. The main reason is that this therapeutic strategy does not use any drug. When one investigates the mechanism of action of a drug, one can study chemical or physical interactions between the drug and a potential drug target (e.g., receptor, enzyme, or DNA). However, because this therapy does not use any drug, physical or chemical interactions with potential drug targets are difficult to measure. In addition, the metabolic environment of cells growing in vitro is extremely different from the metabolic environment of cells living in a whole organism. For example, in vitro, liver and muscle proteolysis cannot supply free AAs to buffer the lack of a particular AA. Any mechanistic insight obtained in vitro will be very difficult to extrapolate to an in vivo situation. Another factor that makes it difficult to understand the in vivo mechanism of action of this anticancer strategy is that it is based on changing the levels of many nutrients simultaneously. In addition, marked anticancer effects are observed with very different combinations of nutrients. Blood tests, together with proteomics and metabolomics analyses in tumor samples and healthy tissues, can provide valuable information on the biological changes elicited by specific artificial diets. When these changes are compared in healthy tissues and tumor samples, one may better understand why this therapeutic strategy affects cancer cells without causing toxicity in healthy tissues. However, it is important to realize that the same pharmacological effect is obtained with very different nutrient compositions. For example, although both diet T2 and diet T14 induce marked anticancer effects in mice with renal cell carcinoma, diet T2 contains high levels of all EAAs including Met, 0% Cys, and high levels of lipids, while diet T14 contains low levels of all EAAs including Met, high levels of Cys, and low levels of lipids. These different combinations of nutrients will lead to different levels of AAs, metabolites, and protein expression profiles in tissue samples. This means that the anticancer activity of this therapeutic strategy cannot be attributed to a particular set of biological changes. Finding common biological changes elicited by artificial diets with very different compositions, and explaining how these changes affect tumor growth without affecting normal tissues, is a highly complex task that is beyond the scope of this manuscript.

The clinical translatability of our anticancer strategy would be simple. The normal diet of cancer patients would be temporally replaced with an artificial diet in which the levels of AAs and other nutrients are precisely controlled. An artificial diet based on diet T18, which has also shown marked activity in mice with other types of metastatic cancers [45], is currently being evaluated as monotherapy in a pilot clinical study in patients with different types of metastatic cancers. Blood tests in these patients will shed light on the metabolic changes elicited by our artificial diets.

## 4. Materials and Methods

### 4.1. Drugs and Reagents

L-asparagine-1-hydrate (Asn, A1668), L-cysteine (Cys, A3694), L-cystine (CySS, A1703), L-serine (Ser, A1708), L-aspartic acid (Asp, A3715), L-proline (Pro, A1707), L-glutamic acid (Glu, A1704), L-tyrosine (Tyr, A3437), L-methionine (Met, A1340), L-alanine (Ala, A1688), L-arginine (Arg, A3675), L-leucine (Leu, A1426), and glycine (Gly, A3707) were obtained from Panreac Química S.L.U. (Barcelona, Spain). Essential amino acid mix, branched-chain amino acids, and L-glutamine (Gln) were purchased from Myprotein (Manchester, England). Cellulose and corn starch were purchased from Farmusal (a local pharmacy, Granada, Spain). Extra virgin olive oil (marketable olive oil developed for Dia Supermarket, Madrid, Spain, 112529) and salmon oil (marketable oil developed for Pets Purest, Wilmslow, England, B06WWFTRXM) were used as a fat source. Sucrose was obtained from local markets. Mineral Mix (AIN-93M-MX, 960401) and Vitamin Mix (AIN Vitamin Mixture 76, 905454) were obtained from MP Biomedicals. Choline bitartrate (450225000), casein (isolated from bovine milk, 276070010), and sunitinib malate (462640010) were purchased from Thermo Scientific Acros Organics (Waltham, MA, United States). We also used doxorubicin (50 mg powder for solution, Farmiblastina, Pfizer, New York, NY, United States, 958314.9), India Ink (Superblack India Ink, Speedball, 33 × 089A, Greenville, CA, United States), and sterile physiological serum for diluting injected drugs (Kin laboratory, Barcelona, Spain, 160407.1). MTT (3-(4,5-dimethylthiazol-2-yl)-2,5-diphenyltetrazolium bromide, A22310005) and SDS (sodium dodecyl sulfate, 1423631209) were obtained from Panreac Química S.L.U. Cell culture reagents were purchased from Biowest (Nuaillé, France) and Thermo Fisher Scientific unless otherwise indicated.

### 4.2. Cell Culture

Mouse renal cell carcinoma cells (Renca, CRL-2947) and human renal cell carcinoma cells (786-O, CRL-1932) were purchased from the American Type Culture Collection (ATCC, Manassas, VA, United States). Both cell lines were maintained in RPMI-1640 medium. Non-malignant human keratinocyte cells (HaCaT, L#300493-4212) were obtained from the Cell Line Service (CLS, Eppelheim, Germany) and they were maintained in Dulbecco’s modified Eagle’s medium (DMEM) [68]. All media were supplemented with 100 U/mL penicillin, 100 μg/mL streptomycin, and 10% fetal bovine serum. All cells were cultured in a humidified 37 °C, 5% CO_2_ incubator.

### 4.3. In Vitro Experiments

In preclinical studies, using human cancer cells, human non-malignant cells, and rodent cells is important to detect selective anticancer activity and possible experimental artifacts caused by species differences in sensitivity to treatments [33,69,70]. Human non-malignant cells (HaCaT), human renal cancer cells (786-O), and mouse renal cancer cells (Renca) were seeded in 96-well plates (8 × 10^3^ cells/well for HaCaT, 5 × 10^3^ cells/well for Renca, and 5 × 10^3^ cells/well for 786-O) in complete DMEM medium. After 24 h, the medium was removed and replaced by amino acid-manipulated media (see details below), complete DMEM medium (controls), or complete DMEM medium with several concentrations of the anticancer drugs sunitinib or doxorubicin. The culture media were changed every three days to avoid exhaustion of nutrients and excessive acidification of the medium. Cells were visualized daily under a microscope and photographed (20× magnification) on the third and seventh days of treatment using a Huawei P9 lite Leica camera (Shenzhen, China) adapted to an inverted microscope. Three days of treatment were sufficient for high doses of sunitinib and doxorubicin to exert a potent cytotoxic effect. Seven days of treatment were necessary to observe a marked cytotoxic effect in cells treated with our AA-manipulated media. Cellular viability was estimated at these times with the MTT assay.

The MTT is a colorimetric assay based on the capacity of viable cells to transform the compound MTT (3-(4,5-dimethylthiazol-2-yl)-2,5-diphenyltetrazolium bromide) into an insoluble purple formazan product; dead cells are metabolically inactive and cannot convert the MTT into the colored product. After treatments, the medium was removed and 125 μL of MTT diluted in medium (1 mg/mL) was added to the wells. The plates were incubated for 2.5 h at 37 °C, 5% CO_2_. Then 80 μL of 20% SDS in 0.02 M HCl were added to the plates, which were incubated overnight at 37 °C. Finally, optical densities were measured at 540 nm on a multiwell plate spectrophotometer reader. Cell viability was expressed as a percentage in relation to controls. Data were averaged from at least three independent experiments.

To prepare the amino acid-deficient media, a concentrated medium was formulated with 8.32 g Dulbecco’s MEM (DMEM low glucose, w/o amino acids, pyruvic acid, USBiological, Salem, MA, United States, D9800-13), 3.7 g sodium bicarbonate (Panreac Química S.L.U., Barcelona, Spain, 141638.1211), 3.5 g glucose (total final concentration of 4.5 g/L, Panreac Química S.L.U, 141341.1211), 100 mL FBS (Biowest, Nuaillé, France, S1810500), 10 mL penicillin-dtreptomycin solution 100× (Biowest, L0022100), and 660 mL of double-distilled water. This solution was sterile filtered using a 0.22-micron membrane filter and then divided into 10 mL aliquots. Each aliquot was supplemented with different combinations of amino acids and completed with water to a final volume of 15 mL. These AA-manipulated media were kept at 4 °C until use. The final concentrations of AAs in these media are specified in Table 1.

### 4.4. Animals

BALB/cAnNRj male mice were purchased from Janvier Labs^®^ (Le Genest-Saint-Isle, France). To allow adequate acclimation, they were housed in our Animal Laboratory Center for at least two weeks prior to the experiments. The animals were kept in standard conditions (24 °C, 70–75% humidity, 12 h light/12 h dark cycle, with ad libitum access to food and water). The mice were fed a control diet (ssniff diet R/M-Z E/R/S; V1724-000, ssniff Spezialdiäten).

All mice were 12 weeks or older at the beginning of the experiments. The experiments were approved by the Animal Ethics Committee of the University of Seville (CEEA-US2018-6/2 and CEEA-US2019-20) and Junta de Andalucía (15/05/2018/090 and 13/11/2020/131). They were conducted under the recommendations of the European Union on animal experimentation (Directive of the European Parliament and of the Counsel 2010/63/EU). All authors of this publication have accredited training in laboratory animal research. Once the experiments started, the animals were watched daily to detect and avoid unnecessary suffering. To follow Animal Ethics Committee recommendations and reduce the number of mice to a minimum, treatments were initially screened using 2–4 mice per group. Additional independent experiments with a higher number of mice were carried out to confirm the anticancer effect of the active diets. Mice receiving the same treatment were merged in the survival curves to facilitate comparison between groups (see table notes and figure captions for details). All experiments included a control group (untreated mice) and a positive control group (sunitinib or anti-PD-1).

### 4.5. In Vivo Experiments

The Renca model was selected because it mimics human renal cell carcinoma and allows the use of immunocompetent mice [71]. Renca cells (passages 7–9) were cultured in 75 cm^2^ flasks until ~80% confluence. The medium was removed and cells were washed twice with a trypsin/EDTA solution. The cells were then incubated with a trypsin/EDTA solution for 3 min at 37 °C to allow the cells to have a rounded shape but without detaching. The trypsin/EDTA solution was aspirated, cells were resuspended in 5 mL of sterile-filtered PBS, and the cell suspension was pipetted up and down to break cell aggregates before adding RPMI-1640 medium supplemented with 2.5% FBS. The cell suspension was centrifuged (5 min at 250 g RT). The medium was then removed and cells were resuspended in warm sterile-filtered PBS. Cells were counted again to ensure that the cell suspension was at the correct density. Finally, a 1 mL syringe (insulin type with a 29 G x 1/2” needle) was filled with 0.2 mL of the working cell suspension, which was injected intraperitoneally into BALB/c mice.

One day before the start of treatments, mice were housed in individual cages to avoid cannibalism. Treatments began 8 days after cancer cell injection when the number of cells inoculated was 10^5^, and 7 days after injection when the number of cells was 1.5 × 10^5^. Treatments with artificial diets lasted at least 4 weeks unless otherwise specified. Treatment with artificial diets consisted of replacing their normal diet with an artificial diet in which the levels of specific components were manipulated. Sunitinib was administered daily in the diet at a dose of approximately 60 mg/kg/day for 28 days. Anti-PD-1 (anti-mouse PD-1 (CD279), clone RMP1–14, BE0146, Bioxcell, Lebanon, NH, United States) was administered intraperitoneally every 4 days for a total of 4 doses. In each dose, mice received a dose of 250 μg anti-PD-1 diluted in pH 7.0 buffer (InVivoPure, IP0070, Bioxcell). Untreated mice were fed the standard diet (ssniff diet R/M-Z E/R/S; V1724-000). All animals were monitored daily and body weights were determined periodically. Mice were sacrificed by cervical dislocation when signs of disease progression were apparent; these signs (e.g., excessive gains and losses in body weight, reduced mobility and curiosity, and/or visible or palpable tumors greater than 15–20 mm) indicated that survival for an additional 48 h was unlikely. A postmortem examination was performed to confirm the cause of death and to observe the extent of the disease. Autopsies confirmed the presence of tumors and similar tumor loads in all euthanized mice (unless otherwise specified or shown).

### 4.6. Diet Preparation and Composition

All artificial diets were created in our laboratory. Diets were prepared by first mixing all solid ingredients until they formed a well-blended dry powder. After the oil was added to the mixture, enough water was added gradually to make a soft dough. The dough was allowed to air dry for approximately 2 h, manually pelleted (approximately 5 g/pellet), left to air-dry for an additional 24 h, and stored at room temperature until use. Diets were usually prepared 2–4 days before starting treatments. The compositions of the experimental diets are shown in Table 2.

Mineral Mix (AIN-93M-MX, MP Biomedical, Eschwege, Germany) constituted 3.5% of the dry diet. One hundred grams of the dry diet contained 1.25% calcium carbonate, 0.875% monopotassium phosphate, 0.098% potassium citrate, 0.259% sodium chloride, 0.163% potassium sulfate, 0.085% magnesium oxide, 0.021% ferric citrate, 0.0058% zinc carbonate, 0.0022% manganese carbonate, 0.0011% copper carbonate, 0.000035% potassium iodate, 0.000035% sodium selenate, 0.000028% ammonium paramolybdate-tetrahydrate, 0.0051% sodium metasilicate-nonahydrate, 0.00095 chromium potassium sulfate-dodecahydrate, 0.0000595% lithium chloride, 0.000284% boric acid, 0.00022% sodium fluoride, 0.00011% nickel carbonate hydroxide, 0.000021% ammonium meta-vanadate, and 0.73% sucrose.

Vitamin Mix (AIN Vitamin Mixture 76, MP Biomedical) represented 1% of the dry diet. One hundred grams of the dry diet contained (mg) thiamine hydrochloride (0.6), riboflavin (0.6), pyridoxine hydrochloride (0.7), nicotinic acid (3), D-calcium pantothenate (1.6), folic acid (0.2), D-biotin (0.02), cyanocobalamin (0.001), retinyl palmitate premix (250,000 IU/g) (1.6), DL-a-tocopherol acetate (250 IU/g) (20), cholecalciferol (400,000 IU/g) (0.25), menaquinone (0.005), and sucrose (972.9).

Casein (bovine casein 27607, Acros Organics, Geel, Belgium) constituted 6% of the dry diets T13–T17. The typical amount (g) of AAs in 100 g and 6 g (shown in brackets) of the casein used in the experiments is: Gln + Glu: 21.7 (1.302), Leu: 9 (0.54), Met: 2.9 (0.174), Phe: 4.8 (0.288), His: 2.6 (0.156), Lys: 7.5 (0.45), Thr: 4.1 (0.246), Iso: 4.3 (0.258), Val: 5.3 (0.318), Trp: 1.2 (0.072), Cys/CySS: 0.7 (0.042), Arg: 3.4 (0.204), Gly: 1.7 (0.102), Ser: 5.7 (0.342), Tyr: 5.2 (0.312), Ala: 2.9 (0.174), Asp + Asn: 6.9 (0.414), and Pro: 10.1(0.606).

Tert-butylhydroquinone (150822500, Acros Organics) was used as an antioxidant for the T13–18 diets, comprising 0.0008% of the dry diet.

The ssniff diet was used as a control diet in this study (SM R/M-S E, 10 mm; V1724-000). The composition, expressed as crude materials, is 21% protein, 7% fat, 4% fiber, 6.2% ash, 33.3% starch, and 4.6% sugar.

### 4.7. Statistical Analysis

Data were expressed as mean ± standard error mean (SEM). Statistical analysis was performed with the GraphPad Prism version 7.0 software. Statistical analysis for the MTT assay was calculated using the nonparametric Mann–Whitney U test. Statistical analysis for the Kaplan–Meier survival curve was calculated using the Gehan–Breslow–Wilcoxon (GBW) test. In all figures and tables, a *p*-value > 0.05 was not considered statistically significant and is not represented by any symbol. A *p*-value ≤ 0.05 was considered statistically significant and was represented with one asterisk (*p* ≤ 0.05).

## 5. Conclusions

Metastatic renal cell carcinoma continues to be an incurable disease for most patients. This work shows that manipulating AA levels with artificial diets has the potential to treat metastatic renal cell carcinoma. Our in vitro experiments revealed that artificial media lacking specific AAs killed renal cancer cells without significantly affecting non-malignant cells. In vivo experiments using a challenging animal model showed that several artificial diets markedly improved the survival of mice with renal cell carcinoma. Mean survivals of mice treated with our diets were higher than in mice treated with sunitinib or anti-PD-1 immunotherapy, which are widely used to treat patients with metastatic renal cell carcinoma. Diet T2 (which lacks Cys, Asn, Pro, Tyr, Glu, and Ser, and contains high levels of Gln and Leu) showed the highest survival improvements in the animals. Diet T18 (a casein-based diet that contains low levels of all AAs except Gln and Leu) also showed anticancer activity in mice with renal cell carcinoma (this work), and in mice with colon cancer [45] and other types of metastatic cancers (our unpublished data). The efficacy and safety of an artificial diet based on diet T18 are currently being evaluated in patients with metastatic cancers in which the standard therapies have failed.

Our in vivo studies provide evidence that the anticancer activity of an artificial diet depends on its whole composition rather than on the levels of a particular AA. Other research groups have previously shown that the restriction of a particular AA is what determines whether a diet is active or inactive. However, our in vivo screening of 18 artificial diets shows that the restriction of a particular AA can have a positive or negative effect on the activity depending on the levels of other AAs. Cys and Met are the clearest examples to support our conclusion. Supplementing Cys in diet T2 reduced its marked anticancer activity, while supplementing Cys in diet T13 markedly improved its anticancer activity (the resulting diet, T14, was even better than sunitinib). In addition, although Met levels were relatively high in all our active diets formulated with free AAs (e.g., diet T2), supplementing similar levels of Met to the casein-based diets (e.g., diet T14) completely blocked the antitumor activity of the diets. Our results warrant additional studies to further evaluate the efficacy and mechanism of action of this simple and inexpensive anticancer strategy.

## 6. Patents

J.M Calderón-Montaño, E. Guillén-Mancina, J.J. Jiménez-Alonso, V. Jiménez-González, E. Burgos-Morón, A. Mate, M.C. Pérez-Guerrero, and M. López-Lázaro are inventors of a patent related to this work licensed to AMINOVITA, S.L., and the University of Seville.

## Figures and Tables

**Table 1:** Amino acid concentrations in the experimental media (g/L).

Amino Acid (g/L)	M0	M1	M2	M3	M4	M5	M6	M7
Leucine	0.528	0.528	0.528	0.705	0.705	0.705	0.705	0.528
Isoleucine	0.096	0.096	0.096	0.096	0.096	0.096	0.096	0.096
Lysine	0.24	0.24	0.24	0.24	0.24	0.24	0.24	0.24
Valine	0.24	0.24	0.24	0.24	0.24	0.24	0.24	0.24
Phenylalanine	0.192	0.192	0.192	0.192	0.192	0.192	0.192	0.192
Threonine	0.16	0.16	0.16	0.16	0.16	0.16	0.16	0.16
Histidine	0.08	0.08	0.08	0.08	0.08	0.08	0.08	0.08
Methionine	0.048	0.048	0.048	0.133	0.133	0.133	0.133	0.048
Tryptophan	0.016	0.016	0.016	0.016	0.016	0.016	0.016	0.016
Glutamine	1	1	1	1	1	1	1	1
Glycine	0.2	-	0.2	0.2	0.2	0.2	0.2	0.2
Aspartate	0.02	-	0.02	0.02	0.02	0.02	0.02	-
Alanine	0.02	-	0.02	0.02	0.02	0.02	0.02	-
Arginine	0.1	-	0.1	-	-	-	-	-
Serine	0.04	-	-	0.04	0.04	-	-	-
Cysteine	0.06	-	-	-	0.06	0.06	-	-
Asparagine	0.05	-	-	-	-	-	-	-
Proline	0.02	-	-	-	-	-	-	-
Glutamate	0.02	-	-	-	-	-	-	-
Tyrosine	0.1	-	-	-	-	-	-	-

**Table 2:** Composition of artificial diets (g/100 g diet).

Artificial Diet	T1	T2	T3	T4	T5	T6	T7	T8	T9	T10
Dietary Constituents										
Casein	-	-	-	-	-	-	-	-	-	-
Glutamine (Gln)	6.00	6.00	6.00	6.00	6.00	6.00	6.00	6.00	6.00	6.00
Leucine (Leu)	6.00	6.00	6.00	6.00	6.00	6.00	6.00	6.00	6.00	6.00
Methionine (Met)	0.60	0.60	0.60	0.60	0.60	0.60	0.60	0.60	0.60	0.60
Phenylalanine (Phe)	2.16	2.16	2.16	2.16	2.16	2.16	2.16	2.16	2.16	2.16
Histidine (His)	0.85	0.85	0.85	0.85	0.85	0.85	0.85	0.85	0.85	0.85
Lysine (Lys)	2.64	2.64	2.64	2.64	2.64	2.64	2.64	2.64	2.64	2.64
Threonine (Thr)	1.80	1.80	1.80	1.80	1.80	1.80	1.80	1.80	1.80	1.80
Isoleucine (Iso)	1.07	1.07	1.07	1.07	1.07	1.07	1.07	1.07	1.07	1.07
Valine (Val)	2.64	2.64	2.64	2.64	2.64	2.64	2.64	2.64	2.64	2.64
Tryptophan (Trp)	0.24	0.24	0.24	0.24	0.24	0.24	0.24	0.24	0.24	0.24
Cystine (CySS)	-	-	-	-	-	-	-	-	-	1.00
Arginine (Arg)	-	1.50	1.50	1.50	1.50	1.50	1.50	1.50	1.50	1.50
Glycine (Gly)	-	1.00	1.00	-	1.00	1.00	1.00	1.00	1.00	1.00
Serine (Ser)	-	-	-	-	-	-	-	-	1.50	-
Tyrosine (Tyr)	-	-	-	-	-	-	1.00	-	-	-
Alanine (Ala)	-	1.00	-	1.00	1.00	1.00	1.00	1.00	1.00	1.00
Aspartate (Asp)	-	2.00	-	2.00	2.00	2.00	2.00	2.00	2.00	2.00
Proline (Pro)	-	-	-	-	-	1.50	-	-	-	-
Asparagine (Asn)	-	-	-	-	1.50	-	-	-	-	-
Glutamate (Glu)	-	-	-	-	-	-	-	2.00	-	-
Olive Oil	14.00	14.00	14.00	14.00	14.00	14.00	14.00	14.00	14.00	14.00
Salmon Oil	-	-	-	-	-	-	-	-	-	-
Choline	0.25	0.25	0.25	0.25	0.25	0.25	0.25	0.25	0.25	0.25
Vitamin Mix	1.00	1.00	1.00	1.00	1.00	1.00	1.00	1.00	1.00	1.00
Mineral Mix	3.50	3.50	3.50	3.50	3.50	3.50	3.50	3.50	3.50	3.50
Sucrose	15.00	15.00	15.00	15.00	15.00	15.00	15.00	15.00	15.00	15.00
Celulose	5.00	5.00	5.00	5.00	5.00	5.00	5.00	5.00	5.00	5.00
Corn Starch	37.25	31.75	34.75	32.75	30.25	30.25	30.75	29.75	30.25	30.75
**Total (g or %)**	100	100	100	100	100	100	100	100	100	100
**Artificial Diet**	**T11**	**T12**	**T13**	**T14**	**T15**	**T16**	**T17**	**T18**		
**Dietary Constituents**										
Casein	-	-	6.00	6.00	6.00	6.00	6.00	6.00		
Glutamine (Gln)	6.00	6.00	-	-	-	-	-	5.00		
Leucine (Leu)	6.00	6.00	-	-	-	-	2.50	2.50		
Methionine (Met)	0.60	0.60	-	-	0.50	0.50	-	-		
Phenylalanine (Phe)	2.16	2.16	-	-	-	-	-	-		
Histidine (His)	0.85	0.85	-	-	-	-	-	-		
Lysine (Lys)	2.64	2.64	-	-	-	-	-	-		
Threonine (Thr)	1.80	1.80	-	-	-	-	-	-		
Isoleucine (Iso)	1.07	1.07	-	-	-	-	-	-		
Valine (Val)	2.64	2.64	-	-	-	-	-	-		
Tryptophan (Trp)	0.24	0.24	-	-	-	-	-	-		
Cystine (CySS)	-	-	-	0.50	-	0.50	-	-		
Arginine (Arg)	1.50	1.50	-	-	-	-	-	-		
Glycine (Gly)	1.00	1.00	-	-	-	-	-	-		
Serine (Ser)	-	-	-	-	-	-	-	-		
Tyrosine (Tyr)	-	-	-	-	-	-	-	-		
Alanine (Ala)	1.00	1.00	-	-	-	-	-	-		
Aspartate (Asp)	2.00	2.00	-	-	-	-	-	-		
Proline (Pro)	-	-	-	-	-	-	-	-		
Asparagine (Asn)	-	-	-	-	-	-	-	-		
Glutamate (Glu)	-	-	-	-	-	-	-	-		
Olive oil	5.00	25.00	-	-	-	-	-	-		
Salmon oil	-	-	1.00	1.00	1.00	1.00	1.00	1.00		
Choline	0.25	0.25	0.25	0.25	0.25	0.25	0.25	0.25		
Vitamin Mix	1.00	1.00	1.00	1.00	1.00	1.00	1.00	1.00		
Mineral Mix	3.50	3.50	3.50	3.50	3.50	3.50	3.50	3.50		
Sucrose	15.00	15.00	15.00	15.00	15.00	15.00	15.00	15.00		
Celulose	5.00	5.00	5.00	5.00	5.00	5.00	5.00	5.00		
Corn starch	40.75	20.75	68.25	67.75	67.75	67.25	65.75	60.75		
Total (g or %)	100	100	100	100	100	100	100	100		

**Table 3:** Survival of mice with renal cell carcinoma treated with diet T2, sunitinib, or anti-PD-1.

Treatment	n	Survival Time (Mean ± SEM; Days)	Survival Improvement vs. Control (Days)	*p*-Value vs. Control
Control	16	35.2 ± 2.5	-	-
Anti-PD-1	9	41.9 ± 4.2	+6.7	0.1070
Sunitinib	7	62.1 ± 7.8	+27.0	0.0032
Diet T2	27	137.8 ± 42.6	+102.6	<0.0001

Data are from several independent experiments. The *p*-value was calculated with the Gehan–Breslow–Wilcoxon test. See text for further details.

**Table 4:** Survival of mice with renal cancer treated with 10 diets based on diet T2.

Treatment	n	Survival Time (Mean ± SEM; Days)	Survival Improvement vs. Control (Days)	*p*-Value vs. Control
Control diet	3	30.3 ± 1.3	-	-
Anti-PD-1	6	43.8 ± 5.1	+13.4	0.0141
T2	7	47.3 ± 4.1	+16.9	0.0016
T3 (−Ala, −Asp)	3	36.7 ± 13.0	+6.3	0.5023
T4 (−Gly)	3	40.3 ± 2.0	+10.0	0.0310
T5 (+Asn)	3	50.3 ± 3.7	+20.0	0.0310
T6 (+Pro)	3	47.0 ± 5.7	+16.7	0.0310
T7 (+Tyr)	3	73.0 ± 14.0	+42.7	0.0310
T8 (+Glu)	3	40.3 ± 7.9	+10.0	0.1120
T9 (+Ser)	3	54.7 ± 7.8	+24.3	0.0310
T10 (+CySS)	3	38.7 ± 0.9	+8.3	0.0310
T11 (5% lipids)	3	55.0 ± 4.0	+24.7	0.0310
T12 (25% lipids)	3	43.0 ± 3.0	+12.7	0.0310

**Table 5:** Survival of mice with renal cell carcinoma treated with diets T13–T17.

Treatment	n	Methionine Supplement (%)	Cysteine * Supplement (%)	Leucine Supplement (%)	Survival Time (Mean ± SEM; Days)	Survival Improvement vs. Control (Days)	*p*-Value vs. Control
Control	4	-	-	-	30.3 ± 3.6	-	-
Sunitinib	4	-	-	-	48.0 ± 3.6	+17.3	0.0308
T13	4	-	-	-	33.3 ± 4.4	+3.0	0.5654
T14	4	-	0.5	-	52.5 ± 12.8	+22.3	0.1893
T15	4	0.5	-	-	30.0 ± 2.1	−0,3	-
T16	4	0.5	0.5	-	29.8 ± 1.9	−0.5	0.8864
T17	4	-	-	2.5	103.0 ± 62.5	+72.3	0.0704

The *p*-value was calculated with the Gehan–Breslow–Wilcoxon test. See Table 2 for diet compositions. See the main text for further details. * Cysteine was supplemented in the form of Cystine (Cys-Cys dimer).

**Table 6:** Survival of mice with renal cell carcinoma treated with diet T18 or sunitinib.

Treatment	n	Survival Time (Mean ± SEM; Days)	Survival Improvement vs. Control (Days)	*p*-Value vs. Control
Control	7	26.9 ± 3.2	-	-
Sunitinib	7	47.8 ± 2.8	+20.6	0.0009
T18	10	41.4 ± 11.0	+14.5	0.0788

The *p*-value was calculated with the Gehan–Breslow–Wilcoxon test. See Table 2 for diet compositions. See the main text for further details.

## Data Availability

Not applicable.

## Supplementary Materials

The following supporting information can be downloaded at: https://www.mdpi.com/article/10.3390/ijms232416132/s1.
