# Supplementary material for: Manipulation of Amino Acid Levels with Artificial Diets Induces a Marked Anticancer Activity in Mice with Renal Cell Carcinoma
Source: Int J Mol Sci. 2022 Dec 17;23(24):16132. doi: 10.3390/ijms232416132 (PMC9783696; doi:10.3390/ijms232416132)
Supplement: Supplementary file 1 [file ijms-23-16132-s001.zip › ijms-1946975-supplementary.pdf]

International Journal of *Molecular Sciences*

Article

Manipulation of amino acid levels with artificial diets induces a marked anticancer activity in mice with renal cell carcinoma

José Manuel Calderón-Montaño[1#], Emilio Guillén-Mancina[1#], Julio José Jiménez-Alonso[1#], Víctor Jiménez-González[1], Estefanía Burgos-Morón[1], Alfonso Mate[2], María Concepción Pérez-Guerrero[1], and Miguel López-Lázaro[1*]

[1] Department of Pharmacology, Faculty of Pharmacy, University of Seville, Sevilla, Spain
[2] Department of Physiology, Faculty of Pharmacy, University of Seville, Sevilla, Spain
* Correspondence: Miguel López-Lázaro. Department of Pharmacology, Faculty of Pharmacy, C/Profesor Garcia Gonzalez 2, Sevilla, 41012, Spain. E-mail: mlopezlazaro@us.es Tel: +34-954-55-63-48, https://orcid.org/0000-0003-2794-1647
# These authors contributed equally to this work

1. SUPPLEMENTARY FIGURES

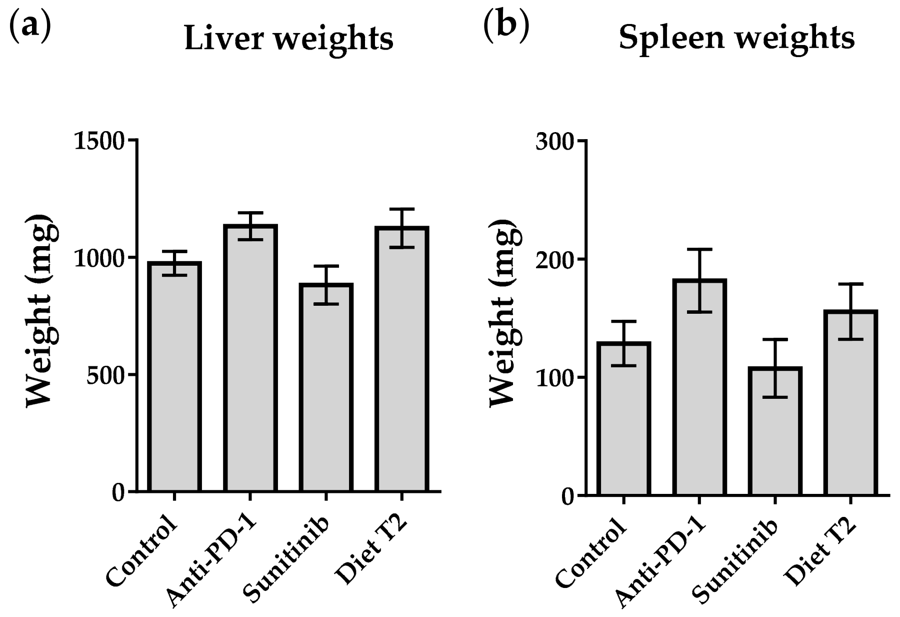

Figure S1. Change in spleen and liver weights of mice treated with diet T2, sunitinib or anti-PD-1 immunotherapy. Liver weights (**a**) and spleen weights (**b**) were measured at different times, when mice reached the endpoint of the experiments (see main text for details).

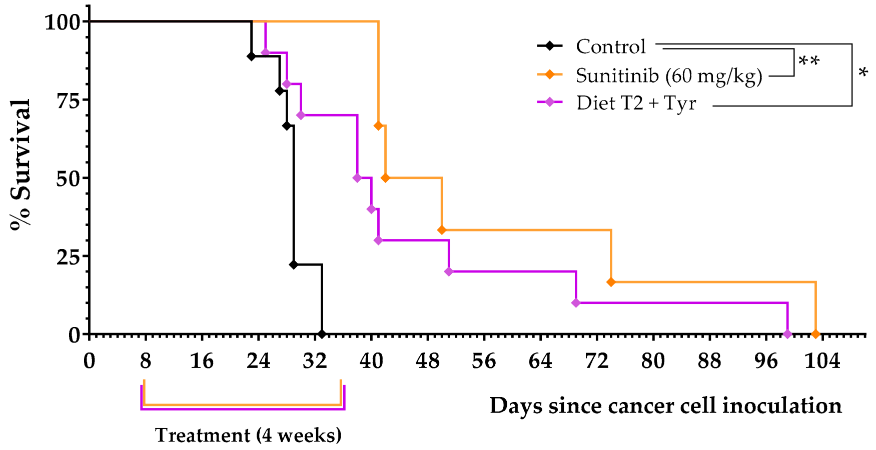

Figure S2. Anticancer activity of diet T2 supplemented with tyrosine in mice with renal cell carcinoma. Treatments started 8 days after the injection of Renca cancer cells. Untreated mice were sacrificed on days 27, 28, 29, 29, 33 and 33. Mice treated with 60 mg sunitinib/kg/day were sacrificed on days 41, 41, 42, 50, 74 and 103. Mice treated with the diet T2 supplemented with 1% Tyr were sacrificed on days 51, 69 and 99; mice treated with the diet T2 supplemented with 1.5% Tyr were sacrificed on days 25, 28, 30, 38, 38, 40 and 41. Statistically significant differences were found for sunitinib and diet T2 + Tyr versus control. Statistical analysis was calculated using the Gehan-Breslow-Wilcoxon test; * indicates $p < 0.05$, ** indicates $p < 0.01$.

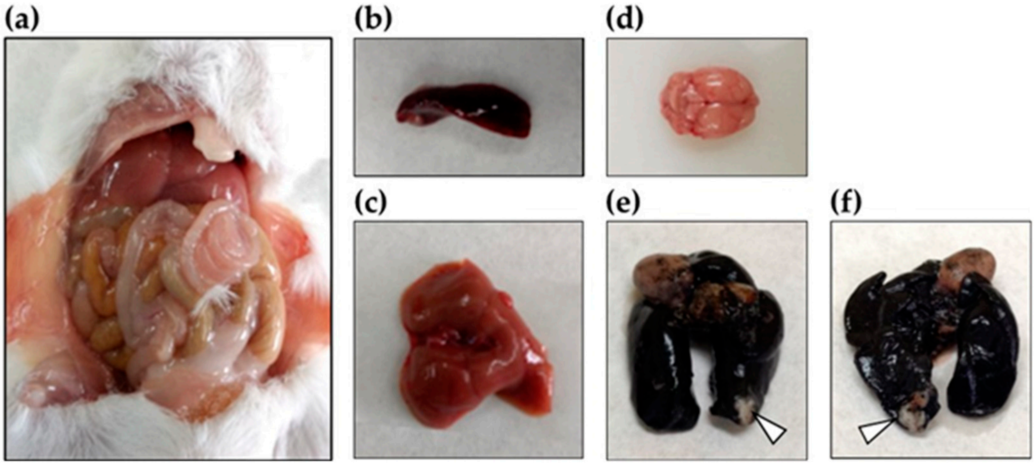

Figure S3. Autopsy images of mouse treated with diet T17 that was sacrificed on day 290. Lack of tumors in the peritoneal cavity (**a**), spleen (**b**), liver (**c**) and brain (**d**). (**e-f**) Front and back view of lungs dyed with India ink with a metastatic tumor. This mouse did not have any symptoms at the time of sacrifice.
